# Control of evaporation by geometry in capillary structures. From confined pillar arrays in a gap radial gradient to phyllotaxy-inspired geometry

**DOI:** 10.1038/s41598-017-14529-z

**Published:** 2017-11-08

**Authors:** Chen Chen, Paul Duru, Pierre Joseph, Sandrine Geoffroy, Marc Prat

**Affiliations:** 1 Institut de Mécanique des Fluides de Toulouse (IMFT), Université de Toulouse, CNRS, INPT, UPS, Toulouse, France; 2 LAAS-CNRS, Université de Toulouse, CNRS, Toulouse, France; 3 LMDC, Université de Toulouse, UPS, INSA, Toulouse, France

## Abstract

Evaporation is a key phenomenon in the natural environment and in many technological systems involving capillary structures. Understanding the evaporation front dynamics enables the evaporation rate from microfluidic devices and porous media to be finely controlled. Of particular interest is the ability to control the position of the front through suitable design of the capillary structure. Here, we show how to design model capillary structures in microfluidic devices so as to control the drying kinetics. This is achieved by acting on the spatial organization of the constrictions that influence the invasion of the structure by the gas phase. Two types of control are demonstrated. The first is intended to control the sequence of primary invasions through the pore space, while the second aims to control the secondary liquid structures: films, bridges, etc., that can form in the region of pore space invaded by the gas phase. It is shown how the latter can be obtained from phyllotaxy-inspired geometry. Our study thus opens up a route toward the control of the evaporation kinetics by means of tailored capillary structures.

## Introduction

Evaporation from capillary structures is encountered in many natural or technological systems and processes such as the evaporation from soils^1^ and from fuel cells^2^, the two-phase heat transport cooling devices used in the electronics cooling industry^3^, evaporation-driven engines^4^, the formation of porous coatings^5^, the drying of porous media^6^, and evaporation with the porous wicks used in several consumer products, such as air-fresheners and insect repellents, for dispensing volatile compounds into the air. Evaporation is also used in microfluidic devices for the exploration of phase diagrams^7^ or the characterization of fluid properties^8^.

In most of these examples, an accurate control of the evaporation rate and of its variation over time is essential. Consider the convective drying of porous media. A simple method to control the drying speed is to adjust the velocity, the temperature or the relative humidity in the surrounding air. However, it is well known^6^ that evaporation is controlled by the external conditions over a limited period only. The internal transfer mechanisms actually play a major role in the overall duration of the process. This suggests the possibility of controlling the evaporation process by acting on the structural properties of the medium, as discussed in, e.g., ref.^9,10^. A simple example is the control of evaporation from soil by varying the properties of a superficial upper layer^11^. A still simpler example is the evaporation from a capillary tube. Making a tube with a polygonal cross section can dramatically reduce the overall drying time compared to that for a circular tube as liquid films become trapped along the internal angles of the tube^12^. In technological devices, heat pipes and two-phase loops^13^ are examples of passive systems where a capillary structure controls the performance. The development of advanced technologies, such as 3D printing and other additive manufacturing techniques offers new possibilities for designing porous structures with properties tailored to specific applications. This reinforces the need for a better understanding of the impact of microstructure on the evaporation process so as to improve the effectiveness of capillary structures used in the applications.

Here, we show how geometry can be exploited in quasi two-dimensional capillary structures to control the drying process. The study is based on one basic idea. Suppose that the evaporation front is located inside a capillary structure, at a distance *δ* from the open surface. The evaporation flux is inversely proportional to this distance. Thus, controlling the drying process comes down to controlling the distance δ and its variation over time. There are two possible ways of controlling *δ(t)*: firstly, through the control of the main liquid – gas invasion front within the capillary structure and, secondly, through the control of capillary liquid films along the solid walls in the regions invaded by the gas phase (gas phase refers to the gas mixture of air and the vapour of the liquid replacing the liquid as it evaporates). The latter will be achieved from phyllotaxy-inspired geometry (*phyllotaxy* ^14^ is the arrangement of leaves on a stem or scales with flowers).

### Experimental set-up

**Figure 1:**
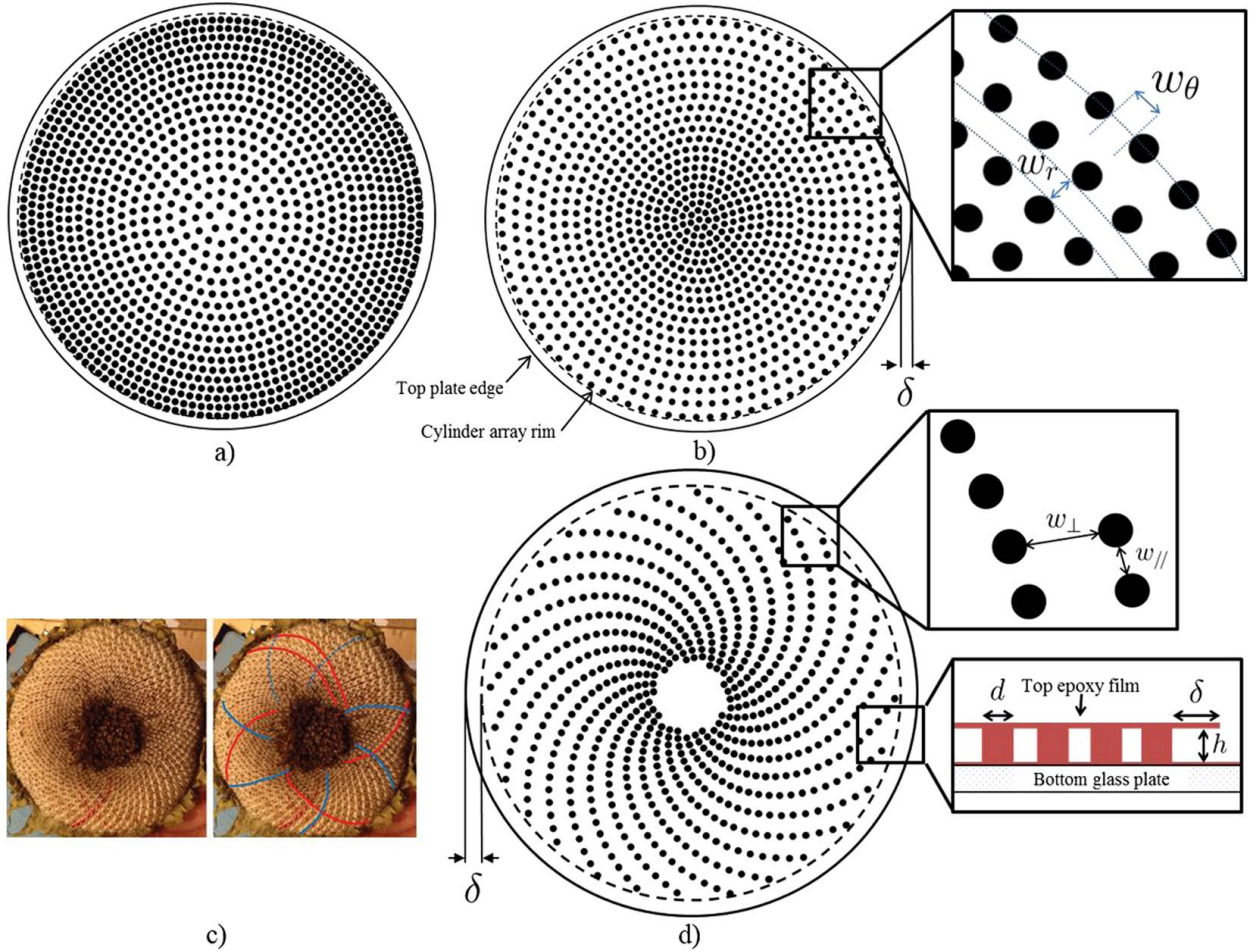
The capillary structures studied: (**a**) unstable system, (**b**) stable system with notations, (**c**) and (**d**) from phyllotaxy to a spiral capillary structure: (**c**) sunflower pistil (reproduced without changes from Fig. 1 in reference ^16^ http://rsos.royalsocietypublishing.org/content/3/5/160091. Credits “The MSI Turing’s Sunflower Consortium” under creative commons license CC-BY 4.0 that can be consulted here: https://creativecommons.org/licenses/by/4.0/), (**d**) our phyllotaxy-inspired capillary structure. The radius, *R*, of the circular top plate is equal to 1.765, 1.62 and 1.74 mm for the stable, unstable and phyllotaxy models, respectively. The outermost pillars are located at about 100 μm from the model rim for the stable and unstable models and at 175 μm on average from the rim for the phyllotaxy model. The pillar diameter, *d*, is 50 μm.

The transparent capillary structures investigated here are arrays of cylindrical pillars confined between two horizontal flat disks (see Fig. 1). Vapour escapes from the outer perimeter of these circular Hele-Shaw cells with pillars. Each system is initially saturated by liquid. The circular top plate is a 20 μm thick, dry epoxy film (DF1020 negative photo-resist^15^) which is laminated on top of the bottom part of the micromodel (after the pillars and bottom plate made in SU8 have been completed by photolithography). The distance, *h*, between the two plates is equal to the diameter *d* of the pillars (*h* = *d* = 50 μm). All the pillars have the same diameter. Other details are given in the caption of Fig. 1. A PCO sCMOS camera with a 2048 × 2048 pixel sensor is used to record top view images of the liquid - gas distribution during the drying process. The experiments are performed with heptane under quasi-isothermal conditions in a room with controlled temperature (*T* = 22 °C).

### Capillary structure design

Two basic features of evaporation in capillary structures were exploited for the design.

#### Pillar arrays in a gap radial gradient

The first was a hierarchical rule. Works on evaporation of a wetting liquid in porous media^17^ have shown that invasion of the pore space by the gas phase when capillary effects are dominant is controlled by the hierarchy in the size of the constrictions of the pore space. The constrictions refer to the narrower passages where the menisci get temporarily pinned. The gas phase preferentially invades the constriction of largest size along the liquid-gas interface. In the pillar arrays, a constriction corresponds to the narrow passage between two neighbouring pillars. Consequently, a simple idea is to act on the spatial organization of the constrictions. This is illustrated here by considering two systems where the pillars are organized in concentric circles. In a first configuration (Fig. 1a), the gap ${w}_{\theta }$ between a pair of pillars along a concentric circle decreases with the distance from the system centre (see Fig. 1b for the definition of ${w}_{\theta }$). The further the pair is from the device centre, the lower the gap. Hence $\frac{d{w}_{\theta }}{dr}$ < 0 in this case, where *r* is the radial coordinate of an *(r, θ)* polar system with its pole at the centre of the device. In contrast, $\frac{d{w}_{\theta }}{dr}$ > 0 for the configuration shown in Fig. 1b, i.e. the closer the pair is to the centre, the lower the gap. These systems are therefore referred to as pillar arrays in a gap radial gradient. As it will be clear from the discussion on the phase distributions presented later, the system with $\frac{d{w}_{\theta }}{dr}$ > 0 is said to be stable because it leads to concentric fronts without fingering whereas the unstable system, with $\frac{d{w}_{\theta }}{dr}$ > 0, leads to (capillary) fingering.

#### Phyllotaxy-inspired capillary structures

Although adjusting the radial gap gradient is a simple way of controlling the drying process, another attractive possibility would be to act on secondary capillary effects. Secondary capillary effects refer to the liquid trapped in geometrical singularities of the pore spaces, such as corners or crevices. When the gas phase crosses a constriction through a piston-like displacement, it is actually rare for the liquid to be totally expelled. A small fraction of liquid remains in the void space invaded in the bulk by the gas phase. The capillary thick films trapped along the corners of a square capillary tube^12^, the liquid bridges at the contact points between particles^18^ or the capillary rings discussed in Ref.^19^ are examples of liquid structures corresponding to secondary capillary effects.

**Figure 2:**
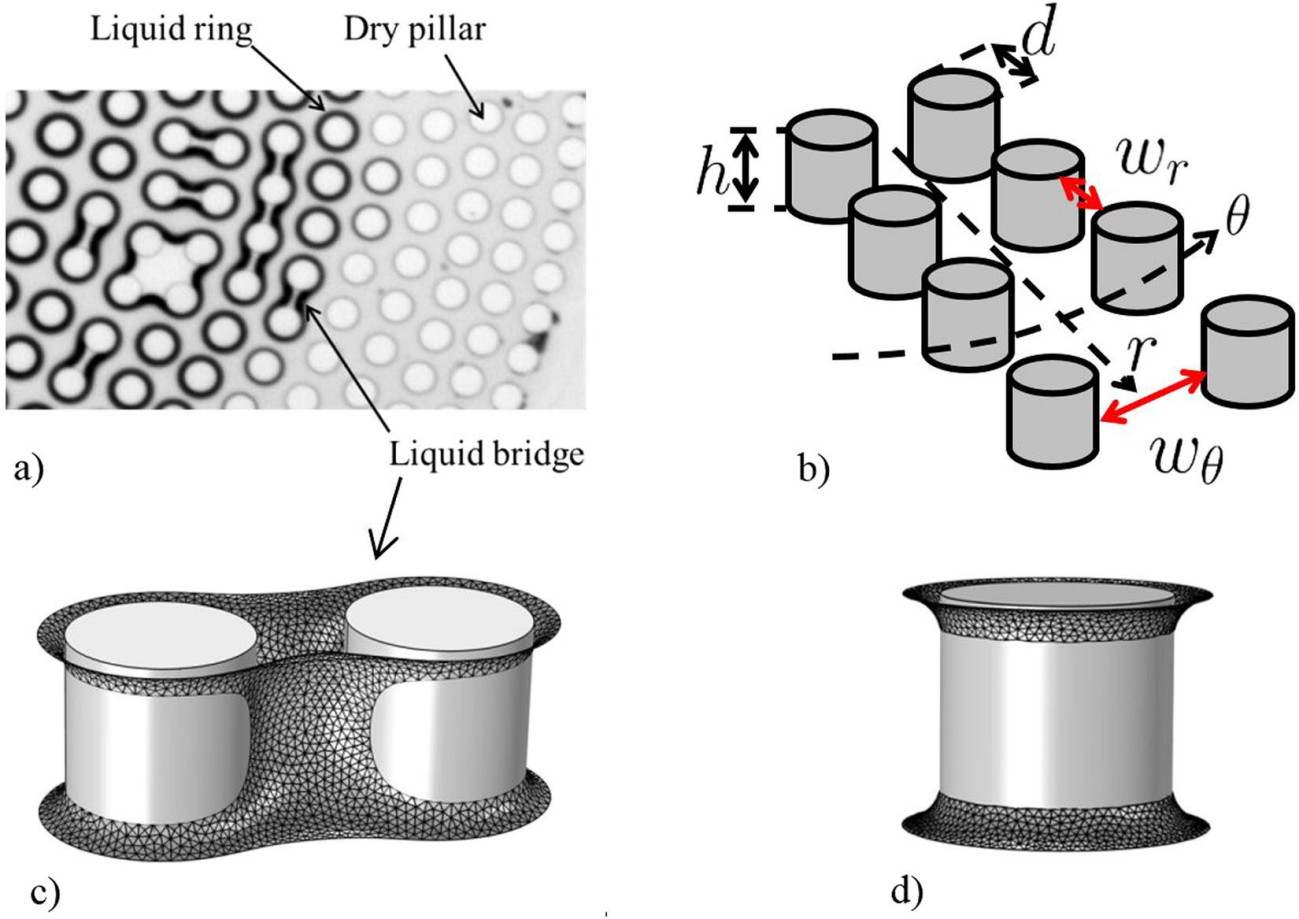
Secondary liquid structures: (**a**) Secondary liquid structures forming during drying in the unstable model. The thick black contours are caused by the presence of liquid-gas interfaces in the horizontal plane of visualization; (**b**) Sketch of pillar array with the notations used; (**c**) Liquid – gas interface of liquid bridge connecting two pillars computed using Surface Evolver^20^, (**d**) Example of computed equilibrium shape of two liquid rings.

As illustrated in Fig. 2a, various secondary liquid structures can form in arrays of pillars confined between two plates, namely liquid rings at the junction between pillars and plates (also in Fig. 2d), liquid bridges between two pillars (also in Fig. 2c), chains of liquid bridges over several consecutive pillars (a chain over four pillars is visible in Fig. 2a) and small liquid clusters (as over the four pillars forming an approximately square pattern in Fig. 2a). Of particular interest are the liquid bridges and notably the chains of liquid bridges.

There are geometrical constraints for liquid bridges to form chains during the drying process. As discussed in the Supplementary Information, the constraint for the cylinder array depicted in Fig. 2b can be expressed as

${w}_{r} < {(\frac{1}{h}+\frac{1}{{w}_{\theta }})}^{-1}$. The question then is how to organize the pillars so as to satisfy this constraint. Furthermore, we wish to obtain long chains of liquid bridges between pillars, possibly longer than a disk radius. Also, we want the distance between two adjacent rows of pillars not to grow too much with the distance from the device centre, so that the pillar density at the periphery remains relatively high. This is important to maintain a high evaporation rate. A simple solution is a phyllotaxy-inspired spatial organization of the pillars. This organisation allows many more pillars to be included within the system and results in a higher pillar density at the periphery than in an arrangement where pillars are simply aligned along radii of the system. Of special interest are spiral arrangements as encountered in the central portion of a sunflower for example (see Fig. 1c). The most common spiral phyllotactic patterns are called Fibonacci patterns because they involve consecutive Fibonacci numbers^21^.

The pillar arrangement shown in Fig. 1d is actually constructed from Fibonacci spirals in such a way that the equivalent of the geometrical constraint ${w}_{r} < {(\frac{1}{h}+\frac{1}{{w}_{\theta }})}^{-1}$ is satisfied. Here, this constraint can be expressed as ${w}_{//} < {(\frac{1}{h}+\frac{1}{{w}_{\perp }})}^{-1}$ where ${w}_{//}$ and ${w}_{\perp }$ are the local gaps between two neighbouring pillars along a spiral and between two neighbouring spirals, respectively (Fig. 1d). As can be clearly seen from Fig. 1d, ${w}_{//}$ < ${w}_{\perp }$, i.e. the distance between a pair of pillars along a spiral is significantly less than the distance between two adjacent spirals in the vicinity of the pair of pillars considered. The geometrical details, such as the parameters of the spirals, the number of spirals, etc. are presented in the Supplementary Information.

## Results

### Phase distributions during evaporation

**Figure 3:**
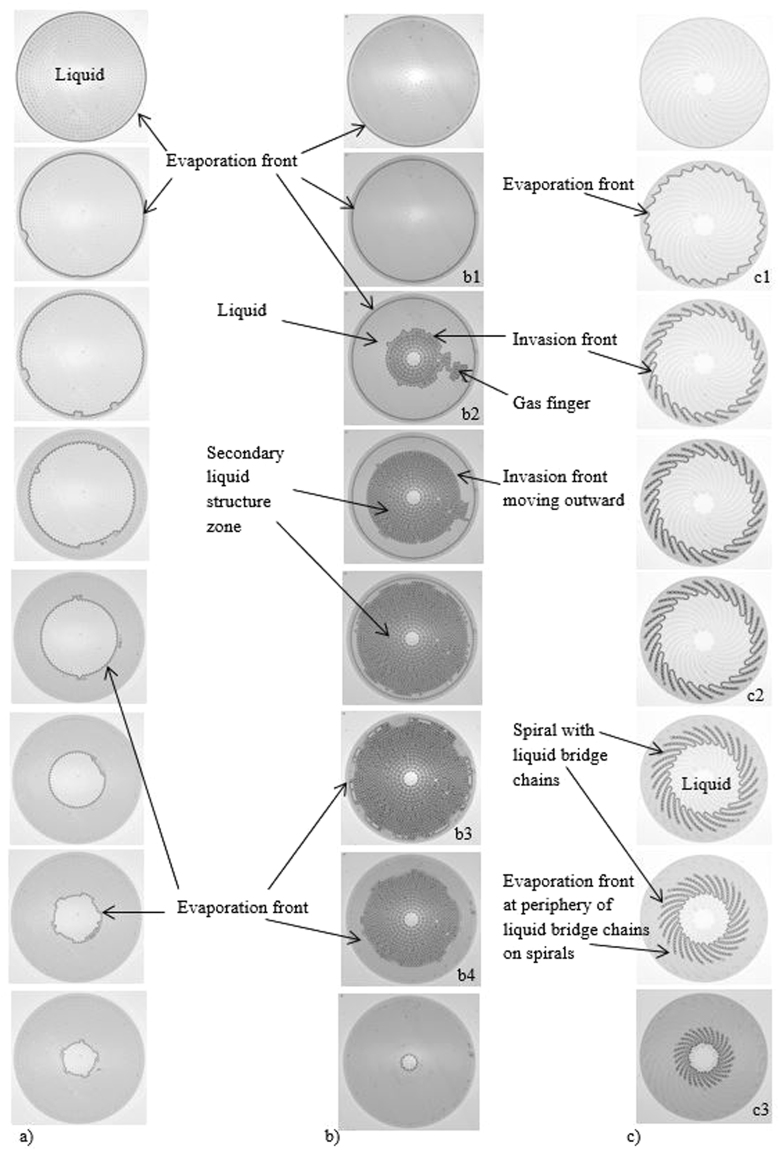
Phase distributions. (**a**) Liquid phase in light grey, darker regions correspond to regions containing gas-liquid interfaces): (**a**) Images of liquid-gas phase distribution during drying in the stable system; (**b**) Images of liquid-gas patterns during drying in the unstable system: (b1) pinning of the evaporation front along the periphery of the outer row of cylinders, (b2) formation of an unstable gas finger and gas zone developing outward from the centre, (b3) collapse of evaporation front pinned on outermost pillars, (b4) last period characterized by the evaporation of liquid trapped within the system at the end of the preceding period; (**c**) liquid –gas phase distribution at different times during drying in the phyllotaxy-inspired system: (c1) evaporation –invasion front pinned onto the outer edge of spirals, (c2) invasion front within the device with chains of interconnected liquid bridges covering all the portions of spirals located in the region invaded by the gas phase, (c3) the tips of the chains of liquid bridges along the spiral have receded into the system. Movies of the three situations (**a**,**b**,**c**) are available online as Supplementary Information.

Figure 3 shows the phase distribution during evaporation for the three systems. The very first period (VFP) is similar in the three systems and corresponds to evaporation until the invasion front is pinned on the outermost row of cylinders. Then, the evolution is markedly different depending on the system considered.

Each system is invaded by the gas phase by successive selection of the constriction with the largest available passage along the liquid – gas interface. As a result, all the azimuthal constrictions between two concentric circles of pillars are invaded before the gas phase can progress again toward the system centre in the stable system. Hence, as shown in Fig. 3a, the invasion is characterized by the successive pinning of the invasion front along each concentric row of cylinders. Since little liquid is temporarily trapped between the invasion front and the system outer edge, the invasion front practically coincides with the evaporation front in this case.

In the unstable system, the gas phase progresses directly toward the centre in the second period, i.e. after the VFP, since the closer the constriction is to the centre, the larger its width is. As depicted in Fig. 3b, the gas invasion in this period is characterized by the formation of a thin tortuous gas finger rapidly connecting the gas phase to the centre of the system.

**Figure 4:**
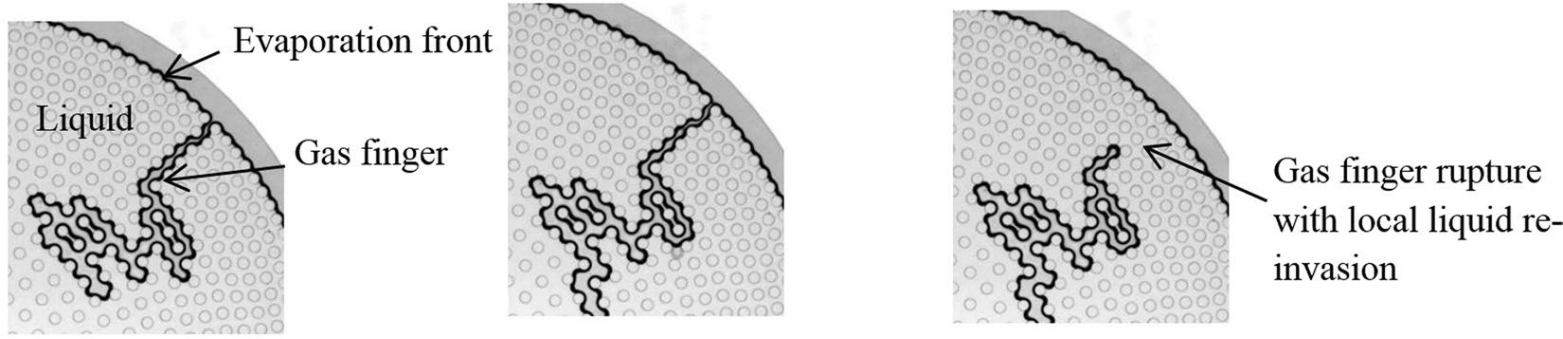
Gas finger rupture in period 2 in the unstable system: formation of an intermittent gas finger, while the evaporation front is pinned along the outer row of cylinders.

As illustrated in Fig. 4, the development of the gas finger is actually unstable and intermittent. In other words, the gas finger advances toward the centre in a series of bursts. A simple explanation for the gas finger pinch-off in the quasi-static limit is provided in the Supplementary Information. A quasi-concentric invasion front forms in the centre while the evaporation front stays pinned on the outermost row of pillars. This invasion front moves outward within the pillar array until it reaches the evaporation front. Secondary liquid structures (small clusters, liquid bridges and rings at the junctions between the pillars and the top and bottom plates as illustrated in Fig. 2a) are present in the region swept by the outward moving invasion front. When the invasion front reaches the pinned evaporation front, then only secondary liquid structures are present in the system. The residual liquid corresponding to the various secondary liquid structures progressively evaporates in the last, slow period of the process as the evaporation front moves inward.

The evolution in the phyllotaxy-inspired device is illustrated in Fig. 3c. As expected since $\frac{d{w}_{\perp }}{ds}$ > 0 where *s* is a curvilinear coordinate along a spiral with origin in the centre of the system, the liquid – gas distribution during evaporation corresponds to a stable pattern. Similarly to the situation in the device with the positive gradient in the gap, the invasion front advances toward the system centre through a series of successive pinnings along an almost concentric alley of cylinders. Because of the organization in spirals, the front is actually scalloped (see Fig. 3c 1). The novel and crucial aspect compared to the stable patterns depicted in Fig. 3a is, as expected, the presence of a chain of liquid bridges along each spiral during a quite significant period of the evaporation process.

As illustrated in Fig. 3c, a liquid bridge chain ceases to develop over the whole spiral branch when the invasion front has advanced sufficiently inside the system. Thus, the tips of the liquid bridge chains stay pinned at the most outer cylinder of each spiral for a while and then start receding into the system. The mechanism leading to the tip depinning is briefly described in the Supplementary Information and is studied in detail in ref.^22^.

By analogy with evaporation in a capillary tube of square cross section^12^, where thick corner films are present, the evaporation front does not correspond to the invasion front but is located at the tips of the liquid bridge chains. Thus, the evaporation front is actually much closer to the system periphery than the invasion front. The physical picture is that the liquid is transported from the receding invasion front through the chains of liquid bridges up to the tip of each spiral where it evaporates. A new period of evaporation begins when both the invasion front and the evaporation front move inward. A very short last period (not shown in Fig. 3c) corresponds to the evaporation of the residual liquid still present along a fraction of each spiral after the invasion front has reached the centre of the device. As can be seen in the video, this very last phase is characterized by a relatively big Haine’s jump where the liquid in the central region of the system is suddenly sucked off into the adjacent pillar regions of higher capillary suction capacity and forms a ring. This is so because no capillary equilibrium is possible between the central region with no pillar and the adjacent pillar regions.

### Evaporation kinetics

**Figure 5:**
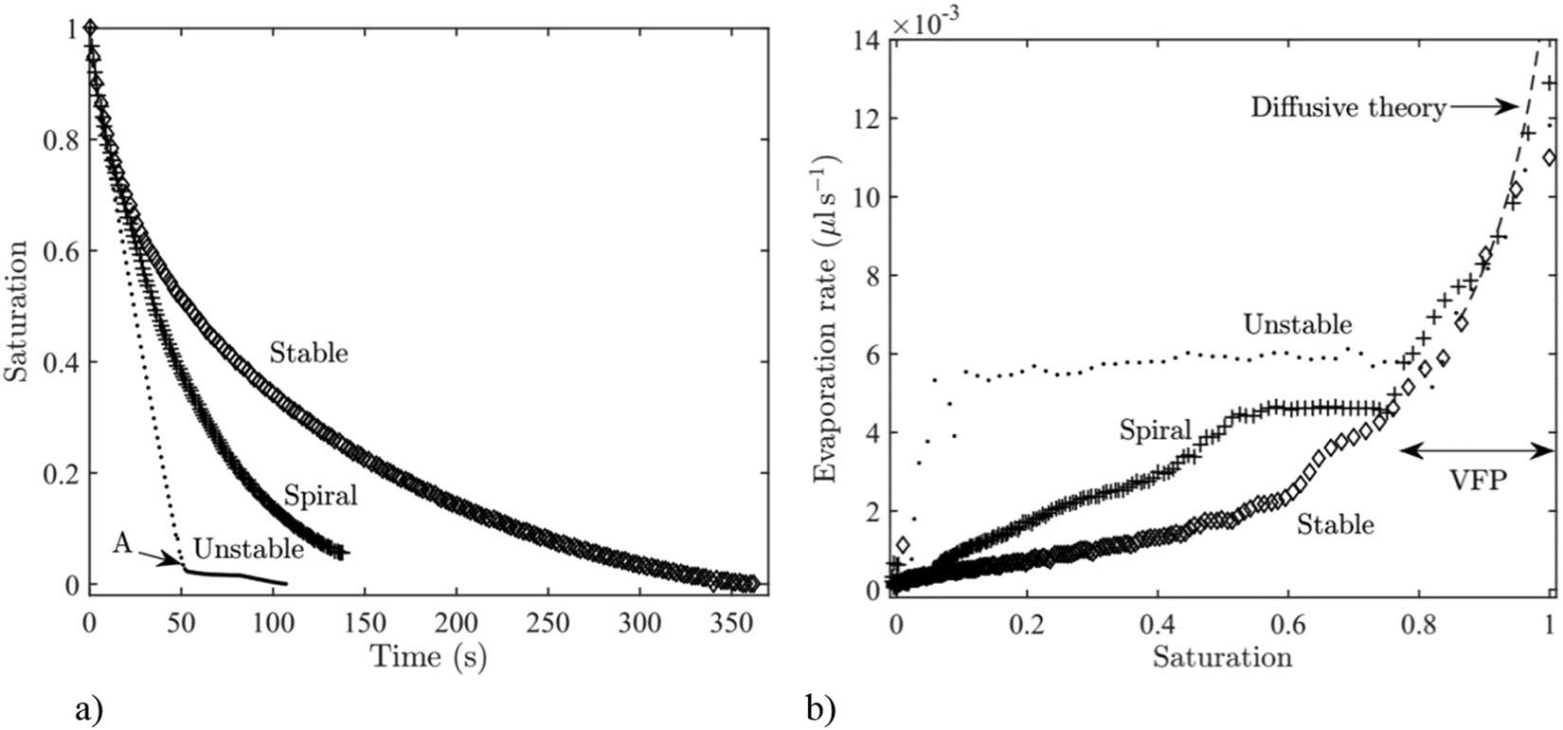
Evaporation kinetics (**a**) liquid saturation as a function of time for the stable, unstable and phyllotaxy (spiral) systems, (**b**) evaporation kinetics for the three systems. The dashed line corresponds to the purely diffusive evaporation theory in a circular Hele-Shaw cell^8^ (see Supplementary Information).

The evaporation curves (liquid saturation as a function of time) and evaporation kinetics (evaporation rate versus the liquid saturation) are plotted in Fig. 5. The saturation *S* is the volumetric fraction of the void space occupied by the liquid phase. Hence, *S* = *1* when a given system is full of liquid and *S* = *0* when it is full of air. The saturation is obtained by image processing. This method for determining the saturation has been validated against gravimetric measurements on similar but larger systems^22^. As for a porous medium^23^, different drying periods are distinguished. The curves are similar for the three systems in the very first period (VFP) when the liquid evaporates until the front is pinned on the outermost row of cylinders. As discussed in the Supplementary Information, simple modelling of the VFP confirms that the transport is by vapour diffusion between the evaporation front and the external air.

Based on the patterns, major differences in the drying kinetics are expected depending on the sign of $\frac{d{w}_{\varphi }}{dr}$ or the presence or absence of corner flows.

Since the evaporation front remains pinned on the outermost row of pillars over a long time in the unstable system, a long constant rate period (CRP) is obtained as illustrated in Fig. 5b. Then, there is a last period characterized by a rapid decrease in the evaporation rate. This last period starts at a low liquid saturation and corresponds to the evaporation of the secondary liquid structures trapped at the end of the CRP.

In contrast, the evaporation rate decreases continuously with the stable system and rapidly becomes smaller than in the unstable system as the dry diffusive zone develops.

As a result, a much shorter overall drying time is obtained with the unstable system compared to the stable one. This is illustrated in Fig. 5a. The point *A* (kink in the curve *S(t)* for the unstable system) is reached in about 50 s. This time corresponds to the collapse of the pinned peripheral evaporation front (Fig. 3b3). Evaporating a similar mass in the stable system takes about 250 s. Thus, the unstable configuration reduces the evaporation time by a factor of about 5.

As expected and illustrated in Fig. 5b, the phyllotaxy-inspired device leads to a quasi-constant evaporation rate period (CRP) since the liquid bridge chains remain attached at the tips of spirals for a long time. The evaporation rate in this period is comparable with that of the CRP for the unstable arrangement. It is clear from Fig. 3 that the distance between the pinned evaporation front and the outer edge of the device is less with the unstable system (see also the caption of Fig. 1). This explains why the evaporation rate in the CRP is greater with the unstable system than with the phyllotaxy-inspired device. The existence of the CRP clearly indicates that the fact that the liquid is only localized at the spiral tips and does not fill the gap between the spirals has a slight impact on the drying rate. This is a classical feature of diffusive evaporation from multiple discrete sources when the density of sources is sufficiently high^24,25^.

The evaporation front, which is located at the liquid bridge chain tips, recedes into the system after a while. This leads to the development of a dry zone between the liquid bridge chain tips and the edge of the system, and therefore to a decrease in the evaporation rate. Figure 5b shows that this falling evaporation rate period is observed when the saturation varies from approximately 0.5 to 0. Therefore, the longer the chains of liquid bridges stay attached to the outermost pillar in each spiral, the longer is the period of high evaporation rate.

## Conclusions

Evaporation can be controlled by the geometry of capillary structures. We can distinguish two main control levers. The liquid- gas interface can be first controlled by adjusting the gap gradient through the control of the piston-like displacement within the pore space constrictions. Secondly, the liquid–interface can also be controlled via the secondary capillary effects. However, this is a somewhat subtler affair. It requires a sufficient understanding of capillary invasion mechanisms to be able to develop designs favouring the formation of thick capillary films or chains of liquid bridges.

These results give a framework for the design of systems with properties tailored to specific applications, e.g. microfluidic devices and porous materials. For example, the gradient in the gap corresponds to a permeability gradient for a porous medium. The design of 3D systems favouring secondary capillary effects (films, liquid bridges) is much more challenging because of the geometrical complexity of the liquid – gas interface in 3D. Relying on advanced morphometric approaches^26^ is perhaps a possibility in this direction. Also, rendering the liquid bridges more resistant to break-up^27^ might be an interesting option.

The formation of liquid bridges is common in porous media^18,28^. Considering the relatively simple bridge chains discussed here can also be seen as a step toward the analysis of the significantly more complex arrangements of liquid bridges found in natural porous media.

## Electronic supplementary material

Movie showing the stable configuration (Figure 3a)

Movie showing the unstable configuration (Figure 3b)

Movie showing the spiral configuration (Figure 3c)

Supplementary Information
