# Supplementary material for: Control of evaporation by geometry in capillary structures. From confined pillar arrays in a gap radial gradient to phyllotaxy-inspired geometry
Source: Sci Rep. 2017 Nov 8;7:15110. doi: 10.1038/s41598-017-14529-z (PMC5678088; doi:10.1038/s41598-017-14529-z)
Supplement: Supplementary file 4 — Supplementary Information [file 41598_2017_14529_MOESM4_ESM.pdf]

Supplementary information to the article

Control of evaporation by geometry in capillary structures. From confined pillar arrays in a gap radial gradient to phyllotaxy-inspired geometry

Chen Chen[1,2,3], Paul Duru[1], Pierre Joseph[2], Sandrine Geoffroy[3] & Marc Prat[1*]

[*]correspondence to Marc.Prat@imft.fr

[1]INPT, UPS, IMFT (Institut de Mécanique des Fluides de Toulouse), Université de Toulouse, Allée Camille Soula, F-31400 Toulouse, France and CNRS, IMFT, F-31400 Toulouse, France
[2]LAAS-CNRS, Université de Toulouse, CNRS, Toulouse, France
[3]LMDC, Université de Toulouse, UPS, INSA, Toulouse, France

System porosity. Fluid properties. Details of spiral system

Table 1. Overall porosity of the three systems

System	Number of cylinders	Outer radius (mm)	Overall porosity
stable	994	1.765	0.8
unstable	1257	1.62	0.7
phyllotaxy	661	1.74	0.86

Heptane properties: diffusion coefficient D = 0.72 10^{-5} m^2/s, dynamic viscosity μ = 0.4 10^{-3} Pa.s, density ρ_ℓ = 682 kg/m^3, surface tension γ = 19.95 10^{-3} N/m, contact angle $\theta \approx 0$ on SU8)
The equilibrium vapour concentration of heptane at 22°C is c_e =0.212 kg/m^3. The heptane vapour concentration in the surrounding air is $c_\infty = 0$.

Spiral systems:
The 661 cylinders of the spiral system are arranged along 28 spirals consisting of 23 or 24 individual cylinders. The centre-to-centre distances between two neighbouring cylinders along a given spiral, $w_{//}$, are in the range 110-60 microns. The centre-to-centre distances between a cylinder and its closest neighbour in the neighbouring spiral (in the counter-clockwise direction), $w_\perp$, are in the range 180-70 microns. More details are given in ref. 22 of the main article.

Geometrical constraint for liquid bridge chain formation

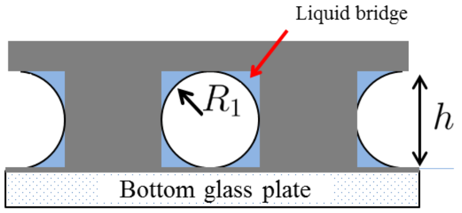

Fig.SI1: Cross section of a liquid bridge in a vertical plane roughly corresponding to the median plane along the cylinders in the radial direction in Fig. 2b.

Let us consider the array of cylinders depicted in Fig. 2b. We wish to approximately determine the total curvature within the bridge immediately after it forms. Since the liquid bridge forms as the result of the piston-like displacement of a bulk meniscus in the radial direction through the constriction between two pillars, the capillary pressure in the liquid bridge is about equal to the capillary pressure threshold necessary for the bulk meniscus to cross the constriction, thus

$$\gamma\left(\frac{1}{R_1}+\frac{1}{R_2}\right) \approx \gamma \cos\theta \left(\frac{2}{h}+\frac{2}{w_\theta}\right) \tag{1}$$

where R_1 and R_2 are the liquid bridge main radii of curvature at the point of its surface considered. Let us suppose that the liquid bridge takes the form of four separated liquid rings around the top and bottom regions of pillars (as depicted in Fig. 2d). We then consider the liquid bridge cross section sketched in Fig.SI1 and make the approximation $R_2 >> R_1$. Assuming a perfectly wetting liquid, we therefore obtain $R_1 \approx \left(\frac{2}{h}+\frac{2}{w_\theta}\right)^{-1}$. Now, suppose we want the bridges to form a chain of interconnected bridges in the radial direction. The requirement is then simply $w_r < 2R_1$, i.e. $w_r < \left(\frac{1}{h}+\frac{1}{w_\theta}\right)^{-1}$. For example, for $h = w_\varphi = d$, this gives $w_r < d/2$. A more accurate constraint requires determining both the isolated bridge curvature as a function of its volume and the constriction invasion capillary pressure threshold. This can be done using Surface Evolver or the method described by Mayer and Stowe in 1965 and 1966.

Mayer, R. P., and R. A. Stowe (1965), Mercury porosimetry - breakthrough pressure for penetration between packed spheres, J. Colloid Sci.,20(8), 893–911.

Mayer, R. P., and R. A. Stowe (1966), Mercury porosimetry: Filling of toroidal void volume following breakthrough between packed spheres, J. Phys. Chem., 70(12), 3867–3873.

Gas invasion finger snap-off (unstable system)

As illustrated in Fig. 4, the period of drying after the VFP in the unstable system is characterized by an interesting process of gas invasion by bursts. The process is as follows. A thin gas finger (see Fig.3b and Fig.4) develops, globally moving toward the centre of the system since the constriction size increases in the direction of the centre. As illustrated in Fig.4, the gas finger is not stable and repeatedly breaks up. A fraction of the gas contained in the gas finger just before the first break-up begins to form a gas cluster near the centre of the system (see Fig.3b) while the remaining shortened branch of the finger recedes toward the outer periphery of the system as a result of a return to capillary equilibrium. Then the gas finger grows again until it reaches the more central gas cluster. A new break-up then occurs resulting in the growth of the detached central gas cluster and in the receding of the shortened finger as after the first break-up. This process repeats itself several times until the central gas cluster has grown sufficiently for the thin gas finger to stabilize. It is also important to note that the evaporation front pinned along the outer concentric row of cylinders is not affected by the gas central invasion mechanism by bursts (see Fig.3b, where it can be seen that the gas –liquid distribution along the most peripheral cylinders does not change).

The reason for the gas finger break-up is explained schematically in Fig. SI2. As in porous materials, e.g. Haines (1930), the gas invasion is envisioned as a succession of quasi-static capillary equilibria. The sudden and rapid event when the phases move from one capillary equilibrium configuration to another is referred to as a Haines' jump. Thus it is during a Haines' jump that the growing gas finger breaks up. The main point to realize is that the capillary equilibrium without constriction refilling (a phenomenon also referred to as "snap-off") is not possible for a gas cluster developing over too long a series of cylinder rows (see Fig. SI2). As sketched in Fig. SI2, corner films form in the corners existing at the contact between the cylinders and the top and bottom plates of the system as the gas finger advances inside the system. Consider only constrictions aligned with the azimuthal direction. Under the condition of capillary equilibrium, we express the fact that the capillary pressure in the corner film is about equal to the capillary pressure threshold necessary for the bulk meniscus to cross the constriction (at the finger tip) through:

$$\gamma\left(\frac{1}{R_1}+\frac{1}{R_2}\right)\approx \gamma\cos\theta\left(\frac{2}{h}+\frac{2}{w_\theta}\right) \tag{2}$$

where R_1 and R_2 are the liquid corner main radii of curvature. Neglecting the radius of curvature in the in-plane direction leads to

$$R_1 \approx \left[\cos\theta\left(\frac{2}{h}+\frac{2}{w_\theta}\right)\right]^{-1} \tag{3}$$

Thus, roughly,

$$R_1 \propto w_\theta \tag{4}$$

The result is that the thickness of the corner liquid films (the greater R_1, the thicker the film) increases as the gas finger advances in regions of wider constrictions. This increase takes place everywhere since the pressure is spatially quasi uniform in each phase under the condition of capillary equilibrium, so also within the constrictions along the gas finger located in the regions where the constrictions are narrower.

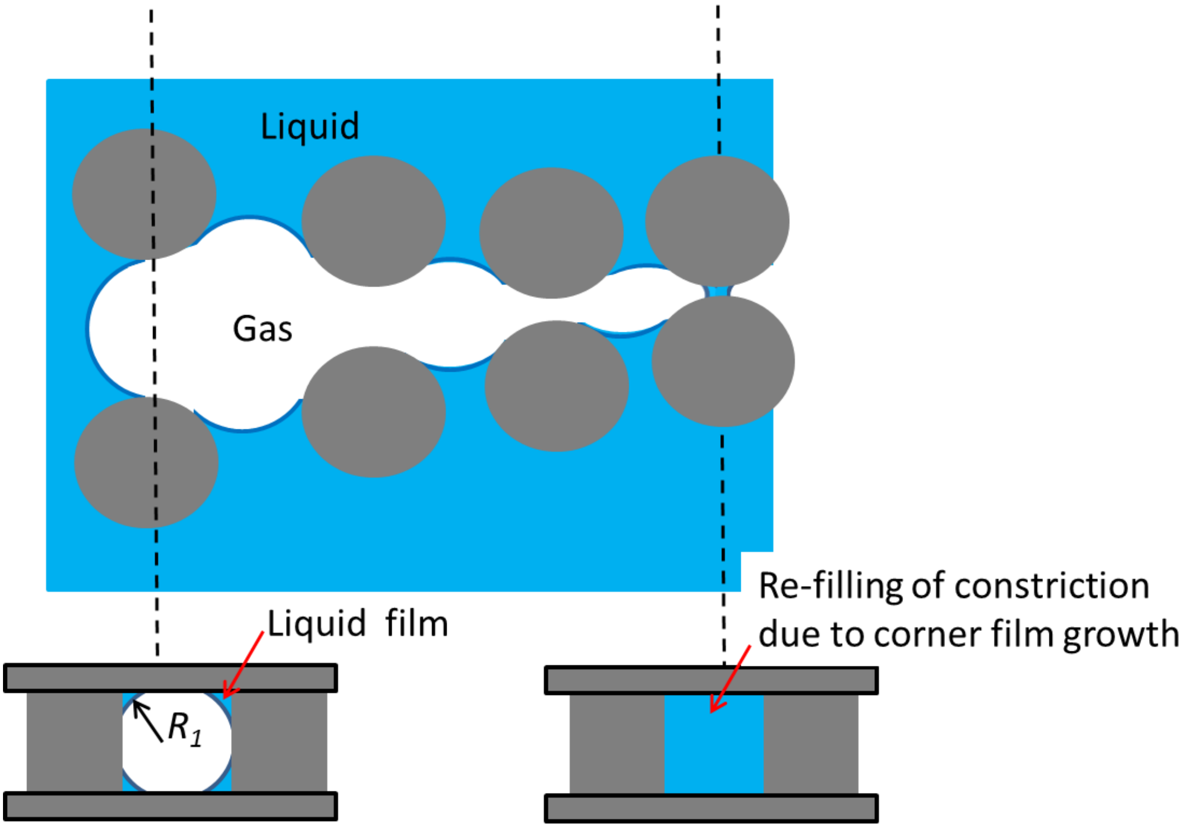

Fig. SI2. Schematic of break-up mechanism of the gas finger as the finger tip advances toward a region of locally lower local capillary pressure threshold.

As a result of the film growth, the liquid corners eventually touch each other in narrower constrictions. Assuming a zero contact angle for simplicity, this must happen in constrictions such that $2R_1 \approx w_\theta$. The "touch event", e.g. Cieplack and Robbins (1990), breaks the capillary equilibrium and leads to gas finger break up via the refilling of at least one narrower constriction. Consistently with this scenario, the images in Fig.4 show that the break-up occurs in the regions of the gas finger where the constrictions are narrowest.

Cieplak, M. and Robbins, M.O. *(*1990*)* Influence of contact angle on quasistatic fluid invasion of porous media. *Phys*. *Rev*. *B*, 41, 11508.

Haines, W. B. , Studies in the physical properties of soil. V. The hysteresis effect in capillary properties, and the modes of moisture distribution associated therewith *The Journal of Agricultural Science* , 20 (01) , 97-116 (1930).

Length of bridge chain on a spiral (tip depinning mechanism)

The mechanisms controlling the length of the liquid bridge chain under quasi-static equilibrium assumptions can be described as follows. A much more detailed analysis is presented in ref. 22. The rationale is somewhat similar to the one presented above as regards the mechanisms leading to the break-up of the gas finger in the unstable system.

Consider the situation depicted in Fig. SI3. The invasion capillary pressure threshold at point A (bulk meniscus) for a perfectly wetting liquid is

$$p_{cr} \approx \gamma\left(\frac{2}{h}+\frac{2}{w_{\varphi A}}\right) \tag{5}$$

Then consider a liquid bridge along the chain of liquid bridges. Under a quasi-static capillary equilibrium condition, we must have

$$p_{cr} \approx \gamma\left(\frac{2}{h}+\frac{2}{w_{\varphi A}}\right) \approx \gamma\left(\frac{1}{R_1}+\frac{1}{R_2}\right) \tag{6}$$

where R_1 and R_2 are the in-depth and in-plane curvature radii of the liquid bridge (see Fig. SI3).

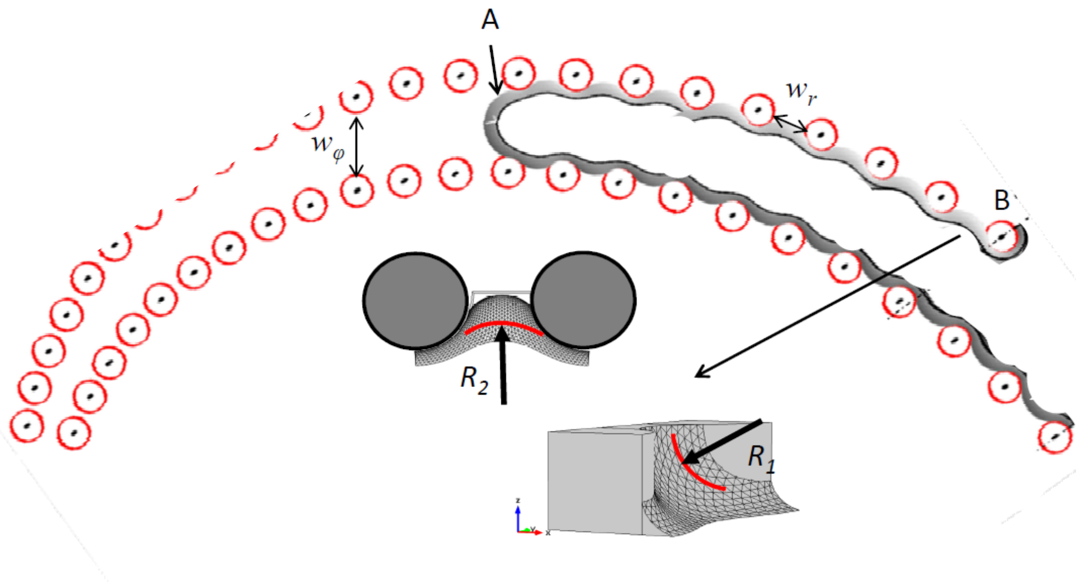

Figure SI3. Chain of liquid bridges (computed using Surface Evolver) with bulk meniscus at point A. R_i is the radius of curvature of the liquid bridge in the narrowest region (constriction) of the liquid bridge.

Since the distance between two adjacent spirals decreases as the bulk meniscus moves further inside the system, $w_{\varphi A}$ decreases as the bulk meniscus A gets closer to the system centre. The result is therefore that the invasion capillary threshold (Eq. (5)) increases as the main meniscus advances between the two spirals. Neglecting R_2 in Eq. (6) in a first approximation, Eq. (6) tells us that R_1 must decrease as the bulk meniscus advances into the system. Thus, the liquid bridges thin out as the main meniscus advances. If we neglect the viscous effects and assume $R_2 << R_1$, then all the liquid bridges thin out in the same way and we cannot explain why the liquid bridge chain breaks up at the tip first. Now, if we make the reasonable assumption that $R_2 \propto w_r$ (i.e. increases with the distance between the two pillars supporting the liquid bridge), we reach the conclusion than $R_1 \propto w_r^{-1}$ according to Eq. (6). Therefore, the chain break-up must take place at the outermost liquid bridge along the chain from capillary equilibrium considerations only.

An additional phenomenon, however, should contribute to the thinning of the liquid bridges. The evaporation process at the liquid bridge chain tips induces a viscous flow within the liquid bridge chains since, for a bulk meniscus to advance, liquid must be transported from the bulk meniscus region to the liquid bridge chain tips, where it evaporates. This viscous flow in the liquid bridge chains is therefore oriented from the bulk meniscus to the liquid bridge tips. It therefore induces a viscous pressure drop ΔP_l within the liquid bridge chains, which

can be expressed as

$$\Delta P_l \approx \frac{\mu}{\rho_\ell G(\ell_s)} \frac{J}{n} \ell_s \tag{7}$$

where J is the evaporation rate, μ is the liquid viscosity, ρ_ℓ is the liquid density, ℓ_s is the length of the bridge chain under consideration along the spiral, n is the number of spirals, and G is the hydraulic conductance (m^4/s) of the bridge chain (see Chen et al. 2015, the reference is given below). As a result, Eq. (6) can be reformulated as

$$\gamma\left(\frac{2}{h} + \frac{2}{w_{\varphi A}}\right) - \frac{\mu}{\rho_\ell G(\ell_s)} \frac{J}{n} \ell_s \approx \gamma\left(\frac{1}{R_1} + \frac{1}{R_2}\right) \tag{8}$$

which expresses the fact that the viscous effect contributes to further thinning out of the liquid bridges located further away from the bulk menisci. Eq. (8) can be used to predict the maximum length of the liquid bridge chain as the bulk menisci recede into the system. However, in addition to the hydraulic conductance G, we also need to know the transition capillary pressure corresponding to the transition between the configurations shown in Fig. 2c and Fig 2d, i.e. the capillary pressure P_{cBK} when the bridge is about to break up (for any further increase in the capillary pressure, the liquid bridge would take the configuration shown in Fig. 2d). This question is addressed in the reference given below and in much greater detail in ref. #21 of the article.

Chen, C. , P. Duru, M. Prat, P. Joseph, S. Geoffroy, Towards the computation of viscous flow resistance of a liquid bridge, Proceedings 8th International Conference on Computational and Experimental Methods in Multiphase and Complex Flow, 20 - 22 April, 2015,València, Spain, 2015.

Variation of evaporation rate during the VFP.
Evaporation in the very first period (VRP) until the front is pinned on the outermost row of cylinders is expected not to be different from evaporation between two disks as studied in (Clément and Leng 2004). Based on the analytical solution proposed in (Clément and Leng 2004), the evaporation rate in the VFP can be expressed as

$$J = 2\pi D h \frac{\Delta c}{\left(\frac{\delta_{ext}}{R} + \ln(R/r_f)\right)} \tag{9}$$

where R is the system radius (numerical values are given in the caption of Fig.1) and r_f is the position of the concentric evaporation front within the system (radial distance from system centre). δ_{ext} represents an external mass transfer characteristic length (assuming that the evaporation rate at the periphery of the system can be expressed as $J = 2\pi R D h \frac{(c_i - c_\infty)}{\delta_{ext}}$ where c_i is the vapour concentration at $r = R$), D is the molecular diffusion coefficient of the vapour, $\Delta c = c_e - c_\infty$, where c_e is the equilibrium vapour concentration of heptane at 22°C (c_e =0.212 kg/m^3) and c_∞ is the heptane vapour concentration in the surrounding air (c_∞ = 0). The only unknown parameter is δ_{ext}. Adjusting δ_{ext} leads to the dashed line in Fig. 5b (and is referred to as "diffusive theory") which is in good agreement with the experimental data. The adjustment leads to δ_{ext} = 79 µm. This is consistent with values of the external length for a capillary tube (ref.12 of the article) indicating that this length is of the order of the tube aperture (thus 50 µm by analogy for our capillary structures).

Clément F. and J. Leng, Evaporation of Liquids and Solutions in Confined Geometry *Langmuir* 2004, *20,* 6538-6541

Movies corresponding to Figure 3:

Three .avi movies corresponding to Figure 3a (Stable_movie), Figure 3b (Unstable_movie), and Figure 3c (Spiral_movie) are available as Supplementary Information. The frame rate of each of these 3 movies is 10 frames per seconds (10 times real time for the stable and spiral cases and 20 times real time for the unstable case).
